# Validity and reliability of Arabic MOS social support survey

**DOI:** 10.1186/s40064-016-2960-4

**Published:** 2016-08-09

**Authors:** Mohamed Dafaalla, Abdulraheem Farah, Sheima Bashir, Ammar Khalil, Rabab Abdulhamid, Mousab Mokhtar, Mohamed Mahadi, Zulfa Omer, Asgad Suliman, Mohammed Elkhalifa, Hanin Abdelgadir, Abdelmoneim E. M. Kheir, Ihab Abdalrahman

**Affiliations:** 1 Soba Centre for Audit and Research (SCAR), Soba University Hospital, University of Khartoum, Alqasr Street, 102, PO Box 321, 11115 Khartoum, Sudan; 2 Department of Pediatrics, Faculty of Medicine, Soba University Hospital, University of Khartoum, Khartoum, Sudan; 3 Faculty of Medicine, Soba University Hospital, University of Khartoum, Khartoum, Sudan

**Keywords:** Arabic MOS survey, Social support, Validity, Reliability, Psychometric properties

## Abstract

We aimed to generate a valid reliable Arabic version of MOS social support survey (MOS-SSS). We did a cross sectional study in medical students of Faculty of Medicine in Khartoum, Sudan. We did a clustered random sampling in 500 students of which 487 were suitable for analysis. We followed the standard translation process for translating the MOS-SSS. We accomplished factor analysis to assess construct validity, and generated item-scales correlations to evaluate the convergent and discriminant validity. We extracted the Cronbach’s α and Spearman Brown coefficient of spit half method to determine internal consistency. We measured stability by correlation between the scores of the MOS survey taken at two different occasions with ten days apart in 252 participants. All items correlated highly (0.788 or greater) with their hypothesized scales. All items in subscales correlated higher by two standard errors with their own scale than with any other scale. Principle component analysis with varimax rotation was conducted on the 19 items and examination of scree plot graphically suggested 4 predominant factors that account for 72 % of variance. It showed high loadings, ranging from 0.720 to 0.84 for items of emotional support, 0.699–0.845 for tangible support, 0.518–0.823 for affectionate support, and 0.740–0.816 for positive social interaction. Cronbach’s alpha for overall MOS scale and subscales indicated high internal consistency. The test–retest correlation showed weak correlation between the test and retest (ranges from 0.04 to 0.104). The Arabic MOS-SSS had high validity and internal consistency.

**Electronic supplementary material:**

The online version of this article (doi:10.1186/s40064-016-2960-4) contains supplementary material, which is available to authorized users.

## Background

Scientists had identified that Social support plays an important role in alleviating the negative effects of mental illness on patients, as well as decreasing distress, improving their self-esteem, quality of life and helping them with dealing with loneliness and despair (Pehlivan et al. 2012). Moreover it had been found that perceived social support decreases mortality and incidence of mental illness (Motamedi Shalamzari et al. 2002). MOS Social Support Survey is one of the most popularly used instruments available in measuring social support. It is a brief, multi-dimensional, self-administered scale initially developed for patients with chronic illnesses including depression. MOS survey is composed of four categories of social support namely (a) Emotional/informational support (the expression of positive affect, empathetic understanding, and the encouragement of expressions of feelings/the offering of advice, information, guidance or feedback), (b) Tangible support (the provision of material aid or behavioral assistance), (c) positive social interactions (the availability of other persons to do fun things with you), and (d) affectionate support (involving expressions of love and affection). The original MOS social support survey was developed and tested for validity and reliability by Sherburne and Stewart in 1985 and found to be highly valid and reliable (Sherbourn and Stewart 1991). The MOS survey was translated and validated for Serbian, French, Portuguese, Chinese, Taiwanese, Malay and Greek languages (Robitaille et al. 2011; Shyu et al. 2006; Soares et al. 2012; Mahmud et al. 2004; Wang et al. 2013).

Correct translation and validation of questionnaires is of paramount importance. The language of questionnaires should be at the level of understanding of the participants. It is essential to word the questions in a way that they can easily be understood by participant and should be according to their educational level and culture, also we should put in our mind the importance of understanding the local context, specific issues and cultural meanings which language carries. If the questions are interpreted differently by the participants it will result in wrong answers and responses will thus be biased (Abdul Momin Kazi 2012). As far as we know, up to the time of writing this manuscript there is no study conducted to validate MOS questionnaire in Arabic language.

If a new questionnaire is to be developed, it should be pilot tested and validated in order to evaluate if it is measuring what it supposed to measure (validity) and if it is doing it reliably. During questionnaire development, its mode of administration should be kept in mind, whether it will be self-administered or interview based and its design and flow should be planned accordingly. A questionnaire undergoes a validation procedure to make sure that it accurately measures what it aims to do, regardless of the responder (Abdul Momin Kazi 2012; Norman and Streiner 2004).

Reliability refers to the repeatability, stability or internal consistency of a questionnaire. One of the most common ways to demonstrate this uses the Cronbach’s alpha statistic. This statistic uses inter-item correlations to determine whether constituent items are measuring the same domain (Jack 1998; Bowling 1997; Bryman and Cramer 1997; Rattray and Jones 2005).

Several studies link social support with psychosocial and physical well-being; nevertheless, the way social support is conceptualized and operationalized differs widely between studies. Some tools focus on structural (social network) or proxy measures such as marital status while others focus on functional aspects of support, such as the Medical Outcomes Study—Social Support Survey (MOS-SSS) questionnaire. Furthermore, the cross-cultural applicability of such measures has not always been established (Nicolaou et al. 2014).

In this study we aimed to obtain a standard translation of the MOS social support survey besides testing the validity and reliability of it. We assessed three aspects of validity; convergent, discriminant, and constructive. In addition, we assessed the internal consistency and stability of the survey over time.

## Methods

We did a cross sectional study in medical students of Faculty of Medicine in Khartoum, Sudan. The Faculty of Medicine has around 1800 medical students who graduate after completing 6 years’ curriculum. We included students who are older than eighteen years. We did a clustered random sampling in students from the second to sixth year and collected 500 questionnaires of which 487 were suitable for analysis. We excluded the first year student because great proportion of them were under eighteen at time of data collection which may interfere with randomization and cause a selection bias.

We followed the standard translation process for translating the MOS survey. A certified translator translated the English version into Arabic. Native English speaker who is fluent in Arabic accomplished backward translation. Committee of two authors who are fluent in English compared the translations and consensus was reached. A pilot data was collected before data collection.

The data collection tool is composed of three questionnaires; the Arabic MOS social support survey, Arabic Depression, anxiety, and stress scale (DAS21), and Arabic WHO quality of life brief WHOQOLB) questionnaire. The validated Arabic version of MOS survey is available as Additional file 1.

1. MOS social support survey (MOS-SSS)

The MOS survey is self-administered and uses five-point answer scales. Self-administered, social support survey that was developed for patients in the Medical Outcomes Study (MOS), a two-year study of patients with chronic conditions. This survey was designed to be comprehensive in terms of recent thinking about the various dimensions of social support. In addition, it was designed to be distinct from other related measures. Empirical analysis indicated that the emotional and informational support items should be scored together, so four functional subscales were derived: tangible support (items 2, 5, 12, 15), affectionate (items 6, 10, 20), positive social interaction (items 7, 11, 14, 18), and emotional or informational support (items 3, 4, 8, 9, 13, 16, 17, and 19). These support measures are distinct from structural measures of social support and from related health measures. They are reliable (all Alphas > 0.91), and are fairly stable over time (Sherbourn and Stewart 1991).

2. The WHO quality of life brief (WHOQOLB) questionnaire

The WHOQOL-BREF produces a quality of life profile. It is possible to derive four domain scores. The WHOQOLB questionnaire is composed of 25 items divided into four domains; physical health, psychological, social, and environment. The four domain scores denote an individual perception of quality of life in each particular domain. Domain scores are scaled in a positive direction (i.e. higher scores denote higher quality of life). The mean score of items within each domain is used to calculate the domain score. Mean scores are then multiplied by 4 in order to make domain scores comparable with the scores used in the WHOQOL-100.

3. The depression, anxiety, and stress (DAS21) questionnaire

The DASS 21 is a 21 item self-report questionnaire designed to measure the severity of a range of symptoms common to both Depression and Anxiety. In completing the DASS, the individual is required to indicate the presence of a symptom over the previous week. Each item is scored from 0 (did not apply to me at all over the last week) to 3 (applied to me very much or most of the time over the past week). The essential function of the DASS is to assess the severity of the core symptoms of Depression, Anxiety and Stress. Accordingly, the DASS allows not only a way to measure the severity of a patient’s symptoms but a means by which a patient’s response to treatment can also be measured. Although the DASS may contribute to the diagnosis of Anxiety or Depression, it is not designed as a diagnostic tool. Indeed, a number of symptoms typical of Depression such as sleep, appetite and sexual disturbances, are not covered by the DASS and will need to be assessed independently. The DAS questionnaire is composed of 3 domains; depression, anxiety, and stress. Each one has 7 items (McDowell 2006).

### Data entry and analysis

Two authors entered the data simultaneously to avoid data entry errors. We accomplished factor analysis to assess construct validity. We generated item-scales correlations to evaluate the convergent and discriminant validity. We extracted the Cronbach’s alpha and Spearman Brown coefficient of spit-half method to determine the internal consistency. We measured stability by correlation between the scores of the MOS survey taken at two different occasions with ten days apart in 252 participants. We used SPSS v22 to analyze data.

## Results

The participants’ age ranged from 18 to 26, with a male to female ration of almost 2:3. A score for each social support scale and the overall scale was computed. We tested the convergent, discriminant, and construct validity, beside the internal consistency and stability.

### Convergent validity

The table below shows the correlation of MOS survey items with their scales, other MOS scales, and other health measures not related to social support.

All the MOS items correlated highly (at least 0.788 or greater) with their hypothesized scales, exceeding our convergent validity criterion (i.e. correlations should be greater than r = 0.30). Item-scale correlations ranged from 0.72 to 0.87 for the tangible support scale, 0.788–0.809 for the affection scale, 0.791–0.882 for the emotional/informational scale, and 0.88–0.892 for the positive interaction scale.

### Discriminant validity

All items in the four functional social support subscales (Table 1) met our criteria of discriminant validity that is, correlated higher by two standard errors with their own scale than with any other social support scale. There was significant association between MOS items and WHOQOLB and DASS-depression items (*P* < 0.05) whereas the correlation of MOS items was weak with DASS, and WHOQOLB items.

**Table 1:** Factors loading

	Rotated component matrix			
	Component			
	1	2	3	4
E1	.772			
E2	.720			
E3	.786			
E4	.839			
E5	.781			
E6	.840			
E7	.824			
E8	.787			
T1		.699		
T2		.843		
T3		.845		
T4		.827		
A1				.823
A2				.824
A3				.518
P1			.764	
P2			.740	
P3			.816	
O1			.761	

Extraction method: principal component analysis

Rotation method: Varimax with Kaiser Normalization

### Construct validity

Kaiser–Meyer–Olkin Measure of Sampling Adequacy was 0.932, and the Bartlett’s Test of Sphericity showed significant results with *P* value less than 0.001. These results indicates the possibility of conducting factor analysis.

**Fig. 1:**
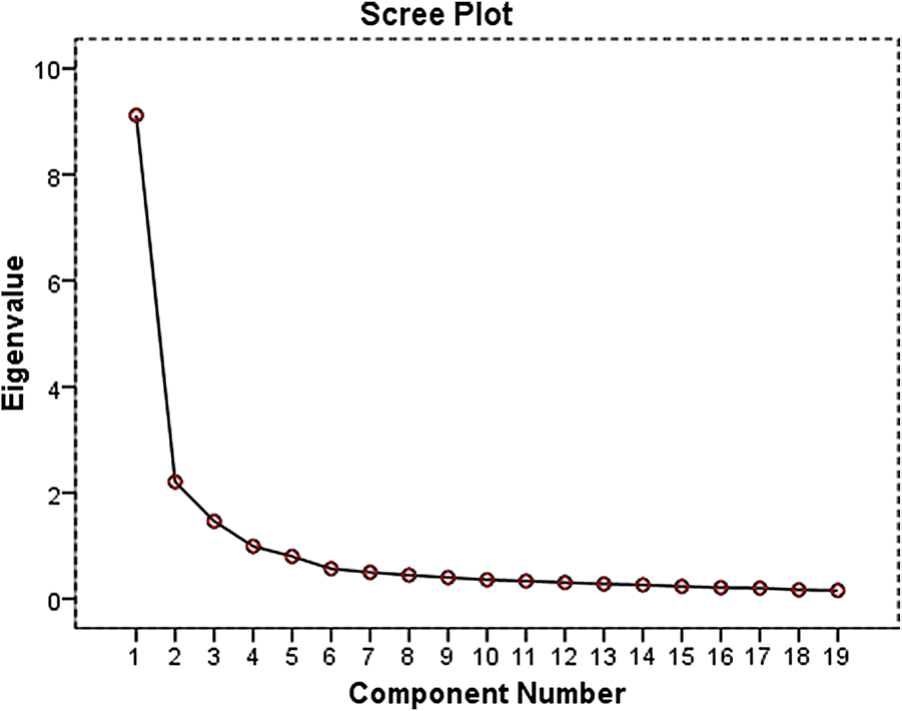
Scree plot demonstrating the Eigenvalues of the elements of Arabic MOS survey

Results of a principal components factor analysis of the 19 support items supported the construction of an overall index. The first un-rotated factor analysis showed high loadings for each of the items, ranging from 0.411 to 0.807 in one factor. Thus, in addition to four subscales, an overall support index which reflects a common higher order support factor can also be constructed. Principle component analysis with varimax rotation was conducted on the 19 items and examination of the initial statistics revealed 3 factors with eigenvalues >1.00 (Table 2). These three factors accounted for 67 % of the variance. The fourth factor has eigenvalue of almost 1.00. The scree plot graphically displayed the eigenvalues of each factor and suggested that there was 4 predominant factors that account for 72 % of variance, as shown in Fig. 1 below.

**Table 2:** Initial eigenvalues and rotation sums of squared loadings

Component	Initial eigenvalues			Rotation sums of squared loadings		
	Total	% of Variance	Cumulative %	Total	% of Variance	Cumulative %
*Total variance explained*						
1	9.117	47.982	47.982	3.150	16.581	16.581
2	2.207	11.617	59.598	1.994	10.493	27.074
3	1.462	7.693	67.292	1.494	7.866	34.940
4	.991	5.215	72.507	1.130	5.946	40.886
5	.800	4.208	76.715	1.073	5.649	46.535
6	.566	2.979	79.695	1.056	5.561	52.096
7	.499	2.626	82.321	1.007	5.300	57.396
8	.447	2.352	84.673	1.002	5.274	62.670
9	.402	2.114	86.787	.975	5.131	67.801
10	.359	1.888	88.674	.913	4.807	72.608
11	.334	1.760	90.434	.911	4.795	77.403
12	.306	1.610	92.044	.865	4.551	81.954
13	.279	1.466	93.510	.858	4.517	86.471
14	.262	1.377	94.887	.634	3.337	89.808
15	.233	1.227	96.114	.528	2.781	92.589
16	.210	1.107	97.221	.476	2.505	95.095
17	.201	1.058	98.280	.428	2.253	97.347
18	.170	.893	99.172	.328	1.725	99.072
19	.157	.828	100.000	.176	.928	100.000

Extraction method: principal component analysis

The rotated component factor analysis showed high loadings for each of the items, ranging from 0.720 to 0.84 for items of emotional support, 0.699 to 0.845 for tangible support, 0.518–0.823 for affectionate support, and 0.740–0.816 for positive social interaction (Table 1).

### Internal consistency

The Cronbach’s alpha for overall MOS scale and subscales was greater than 0.5, which indicates high internal consistency. The results of Spearman Brown coefficient showed in Table 3 supports this finding. Table 4 shows the results of reliability testing.

**Table 3:** Reliability measures (A)

	Alpha	Split half Spearman-Brown coefficient	Stability correlation coefficient
Emotional/info support	0.943	0.922	0.067
Tangible support	0.878	0.82	0.092
Affectionate support	0.651	0.536	0.104
Positive social interaction	0.866	0.866	0.040
MOS overall score	0.929	0.819	0.096

**Table 4:** Reliability measures (B)

	Item-total statistics				
	Scale mean if item deleted	Scale variance if item deleted	Corrected item-total correlation	Squared multiple correlation	Cronbach’s alpha if item deleted
E1	61.84	264.548	.755	.686	.923
E2	61.83	269.712	.696	.644	.924
E3	61.78	268.972	.699	.683	.924
E4	61.77	263.408	.715	.724	.924
E5	62.02	266.153	.658	.604	.925
E6	62.11	261.376	.725	.755	.923
E7	61.93	262.669	.749	.740	.923
E8	62.08	262.569	.722	.678	.923
T1	61.76	270.460	.579	.526	.926
T2	61.18	276.279	.513	.568	.928
T3	61.45	272.053	.537	.659	.927
T4	61.59	269.505	.593	.676	.926
A1	61.72	266.872	.631	.701	.925
A2	61.63	267.891	.625	.700	.925
A3	62.34	260.806	.378	.172	.939
P1	61.49	270.722	.682	.635	.925
P2	61.90	266.108	.683	.621	.924
P3	61.78	269.684	.633	.622	.925
O1	62.08	270.188	.588	.513	.926

We noticed that the only item that if removed the value of Cronbach alpha increases was the last item of affectionate support (A3). In addition, it had the lowest correlation between items and total, which was 0.378, and the scale variance will decrease if it is deleted.

### Stability

The test–retest correlation showed weak correlation between the test and retest (ranges from 0.04 to 0.104) as demonstrated in the table above.

## Discussion

The convergent validity of the Arabic version was high. Similar results were obtained in the validation of the English version of MOS Social Support Survey in 1991. All the MOS items correlated highly (at least 0.788 or greater) with their hypothesized scales, compared to 0.72 or greater, exceeding our convergent validity criterion (Sherbourn and Stewart 1991). Likewise, The Serbian version reported some evidence of independence between measures indicating high convergent validity (Jovanović 2015). In contrast, unsatisfactory item discriminant validity was found in almost half of the items; the item-own subscale correlation was lower than the item-other subscale correlation in the Taiwanese version (Shyu et al. 2006).

Assessment of the discriminant validity also demonstrated high validity. The empirical distinction of the MOS support measures from measures of quality of life, physical and mental health status was confirmed. The MOS scales had weak correlation with the depression, anxiety, stress, and WHOQOLB scales. These findings were consistent with the original MOS survey and Rushidi study which showed weak correlation of MOS survey with other health measures (Sherbourn and Stewart 1991; Mahmud et al. 2004). This indicates that MOS items discriminated well from these measures, supporting their distinction from measures of depression, anxiety, stress, and quality of life. All these findings support the hypothesis that the construction of the overall scale and subscales of this Arabic version seems to be valid.

We assessed the construct validity in terms of factors number and item-scale loading. The confirmatory factors analyses revealed that the number of factors (domains) of the Arabic version was identical into the original scaling of MOS, supporting our scoring of subscales. In addition, an overall support index which reflects a common higher order support factor can also be constructed. Similarly, the confirmatory factor analysis of the French version revealed acceptable fit indices for the 4-factor structure similar to the original one (Robitaille et al. 2011). However, the Taiwanese version validation revealed a two-factor model accounting for 68.98 % of the variance. The first factor (emotional support) accounted for 62.28 % of the total variance, whereas the second factor (tangible support) accounted for 6.7 % (Shyu et al. 2006). Furthermore, exploratory factor analysis of the Portuguese version yielded a three-factor solution, aggregating affection and positive social interaction, and emotional and informational dimensions of social support (Soares et al. 2012). The difference between validation studies was not only limited to number of factors, but also was found in items loading. Analysis of the Arabic version item-scale loading showed high loadings for each of the items in the corresponding factor, ranging from 0.518 to 0.84. This was compatible with the original survey construction research when Sherburne and her colleagues showed that items loading was higher in their supposed domains, where the standardized factor loadings ranged from 0.76 to 0.93 for the tangible support factor, 0.86–0.92 for the affection factor, 0.82–0.92 for the emotional/informational factor, and 0.91–0.93 for the positive interaction factor (Sherbourn and Stewart 1991). In contrary, validation of the Serbian version of MOS survey revealed that only the overall score had high loading (Jovanović 2015). From all of that we can conclude that the item scaling of the Arabic version appears to be appropriate.

Evaluation of the internal consistency using the Cronbach’s alpha and split half method revealed that all subparts of MOS survey are homogenous and measure the same characteristics. This was expected since the MOS survey showed high internal consistency in almost all previous validation studies. The MOS-SSS Chinese Mondrian version had an acceptable internal consistency with Cronbach α coefficients of 0.91 for the overall scale and 0.71–0.84 for the four subscales (Wang et al. 2013). Cronbach’s alpha of the Portuguese MOS was 0.95 for the overall scale, ranging from 0.78 to 0.87 for the five subscales proposed by the original instrument (Soares et al. 2012). The results of the French MOS indicated good internal consistency (Cronbach’s alpha ranged from .90 to .97) and composite reliability (ranging from .93 to .97) for all dimensions of functional social support (Robitaille et al. 2011). Likewise, a study done in Malay by Rushidi, who validated a Malay version of the MOS survey, demonstrated a high internal consistency (r = 0.98) (Mahmud et al. 2004). In the same way, Sherbourn and Stewart (1991) study illustrated that the English version had high internal consistency. The validation of the questionnaire in Greek language showed that all domains had Cronbach’s a value greater than the 0.9 indicating high internal consistency (Nicolaou et al. 2014). This indicates that all items are homogenous and measure the same characteristics even among the different languages.

Stability over time was evaluated using the test–retest method after 10 days interval, and it illustrated weak correlation between the test and retest. Different results were found by the study conducted in Malay which showed a high stability in the questionnaire using the test retest method after 1 week interval with 0.97 coefficient (Mahmud et al. 2004). Sherbourne study showed that the stability of the survey was high over one year with strong correlation (r > 0.7) (Sherbourn and Stewart 1991). The test–retest reliability of the MOS-SSS Chinese mandarin version was generally acceptable with interclass correlation coefficients of 0.89 for the overall scale and 0.74–0.88 for the four subscales (Wang et al. 2013). This low stability might be due to change in the external and internal factors affecting the individuals. However, the possibility of this to occur in 10 days is low. These results may signifies that data of the Arabic version of the MOS survey may poorly reflect participant social support status in one occasion if data was collected at a second occasion. The data of this study cannot provide possible explanation for the low stability, and further research is recommended.

## Conclusions

In short, the Arabic version of MOS survey showed high validity and internal consistency. Further research on its stability may be warranted.

## Additional file

10.1186/s40064-016-2960-4 Arabic MOS survey. The translated items of the English version of MOS survey that were validated (see Tables 1 and 2 for factor loadings, and Table 4 for reliability measures).

## Abbreviations

MOS-SSS: medical outcomes study social support survey
DAS21: depression, anxiety, and stress questionnaire 21 items
WHOQOLB: WHO quality of life Bref questionnaire
