# Supplementary material for: Validity and reliability of Arabic MOS social support survey
Source: Springerplus. 2016 Aug 9;5(1):1306. doi: 10.1186/s40064-016-2960-4 (PMC4978659; doi:10.1186/s40064-016-2960-4)
Supplement: Supplementary file 1 — 10.1186/s40064-016-2960-4 Arabic MOS survey. The translated items of the English version of MOS survey that were validated (see Tables 1 and 2 for factor loadings, and Table 4 for reliability measures). [file 40064_2016_2960_MOESM1_ESM.pdf]

Sample No:………………………..

القيام بمسح الدعم الإجتماعي (MOS social support questionnaire)

يتطلع بعض الناس أحيانا الي الناس الآخرين للرفقة أو المساعدة أوأي أنواع أخري من الدعم. كم من المرات يكون أي من الأنواع التالية من الدعم متاحة لك إذا احتجت لها؟ **ارسم دائرة حول رقم واحد من كل خط.**

	لا أجد أحدا	أجده في قليل من الأحيان	أجده في بعض الأحيان	أجده في معظم الأحيان	أجده في كل الأحيان
**الدعم العاطفي /إعلامي**					
شخص يمكن أن تعتمد عليه ليصغي اليك عندما تحتاج إلي التحدث	1	2	3	4	5
شخص ليقدم لك معلومة تساعدك علي فهم وضع ما.	1	2	3	4	5
شخص ليقدم لك نصيحة جيدة حول أزمة ما	1	2	3	4	5
شخص تثق فيه أو يمكن أن تتحدث معه عن نفسك أو مشاكلك	1	2	3	4	5
شخص تحتاج فعلا إلي نصيحته	1	2	3	4	5
شخص يمكن أن تقاسمه معظم همومك ومخاوفك الخاصة	1	2	3	4	5
شخص يمكن أن تتجه إليه لمقترحات عن كيف تتعامل مع مشكلة شخصية	1	2	3	4	5
شخص يتفهم مشاكلك	1	2	3	4	5
**الدعم الملموس**					
شخص يمكن أن يساعدك في قضاء حوائجك عند اضطرارك لالتزام السرير	1	2	3	4	5
شخص يمكن أن يأخذك إلي الطبيب عند الحاجة	1	2	3	4	5
شخص يمكن أن يعد لك وجباتك عندما تكون غير قادر علي القيام بذلك	1	2	3	4	5
شخص يمكن أن يساعدك في أعمالك اليومية الروتينية عندما تكون مريضا	1	2	3	4	5
**الشعور بالحنان والالفة**					
شخص يظهر لك الحب والعاطفة	1	2	3	4	5
شخص يحبك ويجلك تشعر بأنك شخص مرغوب فيه	1	2	3	4	5
شخص يعانقك ويحتضنك	1	2	3	4	5
**التعامل الاجتماعي الايجابي**					
شخص تقضي معه وقتا طيبا	1	2	3	4	5
شخص تلجأ إليه للاسترخاء والراحة	1	2	3	4	5
شخص تفعل معه شيئا ممتعا	1	2	3	4	5
**بند إضافي**					
شخص تصرف معه أشياء تساعدك علي صرف انتباهك من أشياء أخري	1	2	3	4	5
